# Tele-Rapid Response Team (Tele-RRT): The effect of implementing patient safety network system on outcomes of medical patients–A before and after cohort study

**DOI:** 10.1371/journal.pone.0277992

**Published:** 2022-11-22

**Authors:** Ahmed N. Balshi, Mohammed A. Al-Odat, Abdulrahman M. Alharthy, Rayan A. Alshaya, Hanan M. Alenzi, Alhadzia S. Dambung, Huda Mhawish, Saad M. Altamimi, Waleed Th. Aletreby

**Affiliations:** 1 Critical Care Department, King Saud Medical City, Riyadh, Saudi Arabia; 2 Nursing Department, King Saud Medical City, Riyadh, Saudi Arabia; Clinica Luganese Moncucco, SWITZERLAND

## Abstract

**Background:**

Rapid Response Teams were developed to provide interventions for deteriorating patients. Their activation depends on timely detection of deterioration. Automated calculation of warning scores may lead to early recognition, and improvement of RRT effectiveness.

**Method:**

This was a “Before” and “After” study, in the “Before” period ward nurses activated RRT after manually recording vital signs and calculating warning scores. In the “After” period, vital signs and warning calculations were automatically relayed to RRT through a wireless monitoring network.

**Results:**

When compared to the before group, the after group had significantly lower incidence and rate of cardiopulmonary resuscitation (CPR) (2.3 / 1000 inpatient days versus 3.8 / 1000 inpatient days respectively, p = 0.01), significantly shorter length of hospital stay and lower hospital mortality, but significantly higher number of RRT activations. In multivariable logistic regression model, being in the “After” group decreases odds of CPR by 33% (OR = 0.67 [95% CI: 0.46–0.99]; p = 0.04). There was no difference between groups in ICU admission.

**Conclusion:**

Automated activation of the RRT significantly reduced CPR events and rates, improved CPR success rate, reduced hospital length of stay and mortality, but increased the number of RRT activations. There were no differences in unplanned ICU admission or readmission.

## Introduction

Hospitalized patients can experience several adverse events (AEs), up to and including unplanned admission to the intensive care unit (ICU) or cardiac arrest and death [1]. AEs rates can be as high as 18%, even in countries with well-developed healthcare systems [2]. As many as half of the AEs are preventable [3]. Deteriorating patients in hospitals often experience antecedent deterioration of vital signs for an average duration of 6 hours [1, 4]. If such deteriorations were recognized on time, AEs can be prevented by providing simple interventions to prevent further deterioration [5]. Accordingly, several healthcare authorities have advocated the concept of rapid response teams (RRT), sometimes called rapid response systems (RRS) [6, 7].

Regardless of the structure of RRTs, which varies among countries and according to hospital needs [8, 9], the team remains the efferent (responding) limb of the process and must be activated to provide further assessment and interventions [10]. To be effective, they depend on the afferent limb for activation, which uses specific physiological parameters and predefined thresholds of deterioration to trigger RRT activation, such as the National Early Warning Score (NEWS) or its modified version, Modified Early Warning Score (MEWS) [11, 12]. Since the calculation of these scores and subsequent RRT activation are usually performed manually and depend on staff attentiveness, frequency of measurement, workload, and willingness to call for help [13], there are inevitable delays in RRT activation, which can eventually undermine its effectiveness [1, 14]. Several studies have described delayed RRT activation as one of the most important causes of lack of efficacy [2].

An attempt to decrease delays in RRT activation is by real-time recording of vital signs and automating the calculation of warning scores; however, these modifications did not eliminate the need for human activation of RRT since the alarming vital signs and RRT activation prompts are relayed to ward nurses (either on central screens or paging) [11, 13, 15–18]. Tele-monitoring systems have been designed to overcome this gap by relaying the data of deteriorating patients directly to RRTs, eliminating human factors in activation. Few studies have reported the effectiveness of such systems [15, 16]. However, others have focused on the system’s performance, such as the time to complete vital sign recording, completeness of data, and end-user satisfaction [19].

In this study, we report the effectiveness and patient-centered outcomes of a telemonitoring system that directly relays patient’s vital signs and warning scores to the RRT. We conducted this study under the hypothesis that implementing the system would decrease severe AEs due to early activation of RRT.

## Materials and methods

This was a before-after cohort study comparing prospective data after intervention to retrospective data before. We conducted the study in a large government hospital in the central region of Saudi Arabia (1200 inpatient beds), where the ICU provides outreach services in the form of an RRT to the general ward.

### RRT structure and function

The RRT consisted of an intensivist, two intensive care nurses, and a respiratory therapist. The RRT covers referrals to the ICU by primary admitting specialties, in addition to 48 hours follow up of patients discharged from the ICU regardless of their condition, since our hospital lacks a step-down unit. The main purpose of RRT is the early management of deteriorating patients to stop their deterioration and prevent unplanned ICU admission or cardiac arrest. This could be achieved by simple interventions such as suctioning, administering analgesics, antipyretics, antihypertensives and intravenous fluids, providing supplemental oxygen, and chest physiotherapy. Patients who have already deteriorated significantly and become critically ill are beyond the scope of RRT; such patients require more intensive interventions such as endotracheal intubation or initiation of vasoactive medications, and their ICU admission is advisable. If the RRT interventions fail to prevent further deterioration, the patient is handed over to the mobile team or admitted to the ICU. Accordingly, RRT is not the only source of admission to the ICU from the ward, and the majority of ICU admissions from the ward are through the mobile team, which tends to refer critically ill patients who are beyond the scope of RRT. ICU admissions by RRT were all considered unplanned. During COVID-19 pandemic, all COVID-19 positive patients were admitted to isolation wards under the supervision of ICU, and were discharged to general wards only upon negative swab results, the RRT applied standard infection control precautions when activated throughout the study.

### RRT activation criteria

As per hospital policy, RRT may be deployed if the patient’s vital signs show objective deterioration based on an early alert system, which utilizes the criteria of MEWS, and a patient is eligible for RRT activation with a score ≥ 5. Although a triggering score of 6 was found to be an excellent predictor of ICU admission [12], the cutoff value of 5 for the MEWS score was chosen as an extra precaution to identify deteriorating patients as early as possible (S1 Table). Once activated, the team followed-up the patient daily without the need for reactivation until discharge from their services.

### Procedure and patients

We included data from 8 months between January and August 2020 as the pre-intervention period “Before” group, while the post-intervention period “After” group included data from the last 4 months of 2020 and the first 4 months of 2021 (September 2020 –April 2021). Patient data from both groups were collected from the RRT register and the hospital’s electronic database, and the quality and completeness were supervised by two authors. Recorded data included age, sex, whether the patient was a new referral to RRT, or was being followed-up for 48 h after ICU discharge. The main diagnostic medical category included internal medicine, neurology, nephrology, pulmonology, hem-oncology (surgical patients in the study were not included due to feasibility of implementation in the surgical units), MEWS upon RRT activation, and number of RRT activations per patient (discounting false activations that resulted from connectivity problems with the sensors). We also recorded the length of stay (LOS) of each patient in the ward; however, the reported LOS in this study includes only days in the ward before discharge, ICU admission/readmission, or death. Accordingly, for patients discharged from the ICU, the LOS did not include the period of stay in the ICU. For both groups, the following outcome variables were recorded: ICU admission or readmission, ward cardiopulmonary arrest and subsequent CPR occurrence, and death. Patients transferred to other healthcare facilities were not followed up and were considered alive at discharge.

We included all adult patients (age ≥ 18 years) in the medical wards, followed by RRT, regardless of whether they had new activation or follow-up after ICU discharge. We excluded the surgical wards, patients not followed by RRT, and patients labelled as “Do Not Resuscitate.” The exclusion of surgical patients was due to feasibility, as we had limited number of sensors and bedside devices. All patients were enrolled in the study only once to maintain data independence. Accordingly, we accounted for only the first episode of RRT activation, even if there were subsequent episodes.

### Outcomes

The primary outcome measures were the occurrence of CPR, CPR rate, and CPR success rate. Successful CPR was defined according to our hospital’s policy as return of spontaneous circulation (ROSC) for at least 20 min [20]. The secondary outcomes included ICU admission, number of RRT activations per patient (including first-time activation and subsequent visits), and medical-ward hospital mortality.

### Intervention

We applied Masimo Patient SafetyNet ^TM^ (Masimo, Irvine, CA) to all patients of the “After” group. The system is composed of sensors connected to bed-side devices that non-invasively monitor patient’s vital signs, wirelessly send data to a server capable of simultaneously registering 200 patients, and display real-time information (updated every 5–30 min according to the patient’s condition) on a central screen that can display data of up to 40 patients. A previously validated [21, 22] RD rainbow SET-2 Neo ® sensor was used to capture the heart rate, respiratory rate, and oxygen saturation, whereas blood pressure was measured using a root machine (inflatable arm cuff). The system also included “Replica,” a mobile application that allows authorized personnel to view patient data and receive alarms on smartphones. The software was pre-programmed to alarm visually and audibly when any of the vital signs included in the MEWS calculation (systolic blood pressure, heart rate, respiratory rate, and peripheral oxygen saturation) exceeded its upper or lower limits, as well as when the total MEWS was ≥ five based on automated calculation, with level of consciousness and temperature as a manual input by the ward nurse every 4 hours.

Masimo provided the Patient SafetyNet TM system; however, they were not involved in the data collection, analysis, or drafting of this study. The study was approved by the local institutional review board with waiver of consent (registration number: H1RI-07-Jan20-02), as the application of the tele-RRT system was considered a performance improvement project, and registered at researchregistry.com # 7898. This study followed the ethical principles outlined in the Declaration of Helsinki.

### Statistical method

Continuous data are summarized as mean ± standard deviation (SD) and discrete data as frequencies and percentages (n, %). Continuous data were compared using Student’s t-test or Wilcoxon rank sum test as appropriate, and discrete data by chi-square test or Fisher’s exact test, as appropriate. We calculated the CPR rate as the number of events divided by inpatient days in ward times 1000 (unit is CPR / 1000 inpatient days).

We fitted a multivariable logistic regression model of predictors of CPR, using the backward deletion method to retain variables in the model if their p values were > 0.1. The goodness of fit of the model was evaluated using the Hosmer–Lemeshow test (well fitted if p > 0.05), and presented using area under the receiver operator characteristics curve of probability prediction of the model along with the percentage of correctly classified cases. We also explored the fulfillment of logistic regression assumptions.

As a sensitivity test for the primary outcome, we fitted a Kaplan–Meier curve for survival of both groups and reported the p-value of the log-rank test.

All statistical tests were two-tailed and considered statistically significant if the p-value was < 0.05, without correction for multiple testing; hence, their results could only be considered exploratory. The commercially available statistical package STATA ® was used for analyses (StataCorp. 2015. Stata statistical software: Release 14. College Station, TX, StataCorp LP).

## Results

**Fig 1:**
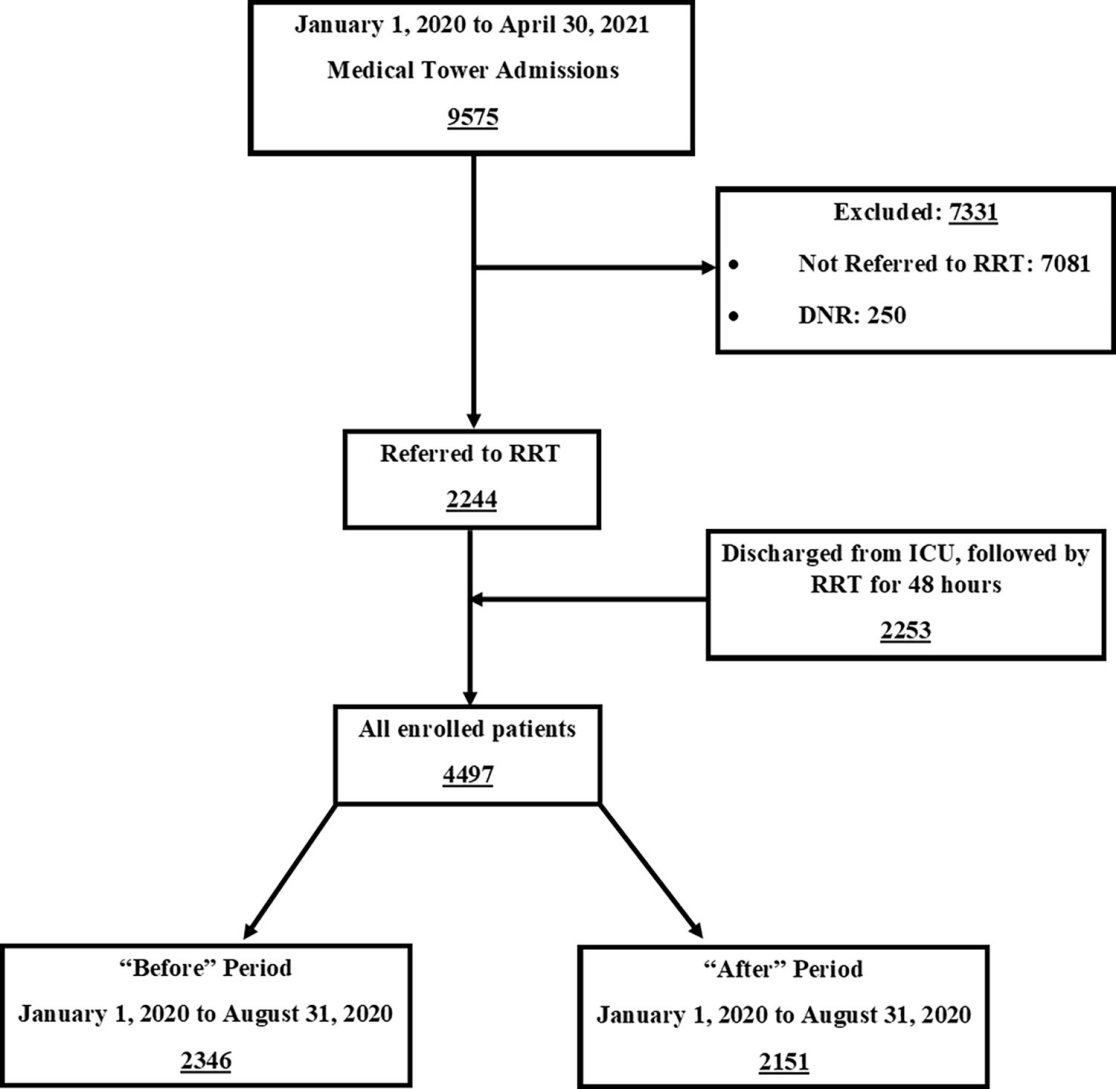
Patients’ enrollment flow diagram.

The “Before” and “After” groups included data of 2346 and 2151 patients respectively (Fig 1). Notably, the DNR rates were similar in both study periods. Table 1 shows the demographic and clinical characteristics of both the groups. There were no differences in sex distribution, percentage of patients discharged from the ICU, or MEWS at the time of RRT activation. However, the “After” group had a higher average age compared to “Before” group. In both groups, the predominant diagnostic category was internal medicine, without differences in the distribution of all categories between the groups (S1 Fig).

**Table 1:** Demographic and clinical characteristics of study groups.

	Before (n = 2346)	After (n = 2151)
**Age (yrs.)**	52 ± 4.1	52.5 ± 3.7 *
**Gender**		
**Males**	1220 (52%)	1108 (51.5%)
**Females**	1126 (48%)	1043 (48.5%)
**Discharged from ICU**	1161 (49.5%)	1092 (50.7%)
**Diagnostic Category:**		
**Medical**	932 (39.7%)	848 (39.4%)
**Nephrology**	600 (25.6%)	549 (25.5%)
**Neurology**	496 (21.1%)	458 (21.3%)
**Pulmonology**	245 (10.4%)	224 (10.4%)
**Hem-Oncology**	73 (3.2%)	72 (3.4%)
**MEWS (SUM)**	5.5 ± 0.8	5.6 ± 0.8

*Wilcoxon rank sum test due to non-normal distribution of data.

¶ p < 0.001

ICU = intensive care unit.

Table 2 shows the primary and secondary outcomes. “Before” group had 78 episodes of CPR, and total in-patient days of 20,510. This accounts for a CPR incidence of 3.3% and a CPR rate of 3.8 / 1000 inpatient days (95% confidence interval (CI): 3–4.7). Whereas, “After” group had a total of 17,945 inpatient days and 42 CPR episodes, which yielded CPR incidence of 1.95% and CPR rate of 2.3 / 1000 inpatient days (95% CI: 1.7–3.2). CPR incidence in the “After” group was significantly lower than “Before” group (p = 0.01). The CPR success rate was significantly higher in the “After” group compared to “Before” group (59.5% vs. 38.5%; p = 0.04). There was no statistically significant difference in the percentage of ICU admissions between the groups. In contrast, the “Before” group had a significantly higher average LOS and fewer RRT activations. Moreover, 3% of RRT activations were false due to connectivity issues which could not be judged except at the patient’s bedside. We report only the true ones, S2 Fig shows the triggers of RRT activation in both groups. The overall hospital mortality of medical wards was significantly lower in the “After” period (5.4% vs. 4%, 95% CI: 0.6–2.2; p < 0.001).

**Table 2:** Primary and secondary outcomes.

	Before (n = 2346)	After (n = 2151)	95% CI of difference	P value
***Primary Outcomes***				
CPR (n,%)	78 (3.3%)	42 (1.95%)	0.4% to 2.3%	0.01
Total in patient days	20510	17945		
CPR Rate: CPR / 1000 patient days	3.8 (95% CI: 3–4.7)	2.3 (95% CI: 1.7–3.2)	0.3–2.6	0.01
CPR success rate	30/78 (38.5%)	25/42 (59.5%)	1–39.4%	0.04
***Secondary Outcomes***				
ICU Admission	49 (2.1%)	50 (2.3%)	-2% to 4%	0.5
aLOS (days)	8.7 ± 3.4	8.3 ± 3	0.2 to 0.6	< 0.001*
Number of RRT activations	20 ± 7	23.7 ± 9.4	3.2–4.2	< 0.001*
Medical wards’ Hospital mortality	304/5623 (5.4%)	251/6205 (4%)	0.6 to 2.2	< 0.001

*Wilcoxon rank sum test due to non-normal distribution of data.

CPR = cardiopulmonary resuscitation, ICU = intensive care unit, aLOS = average length of stay, RRT = rapid response team, CI = confidence interval.

**Fig 2:**
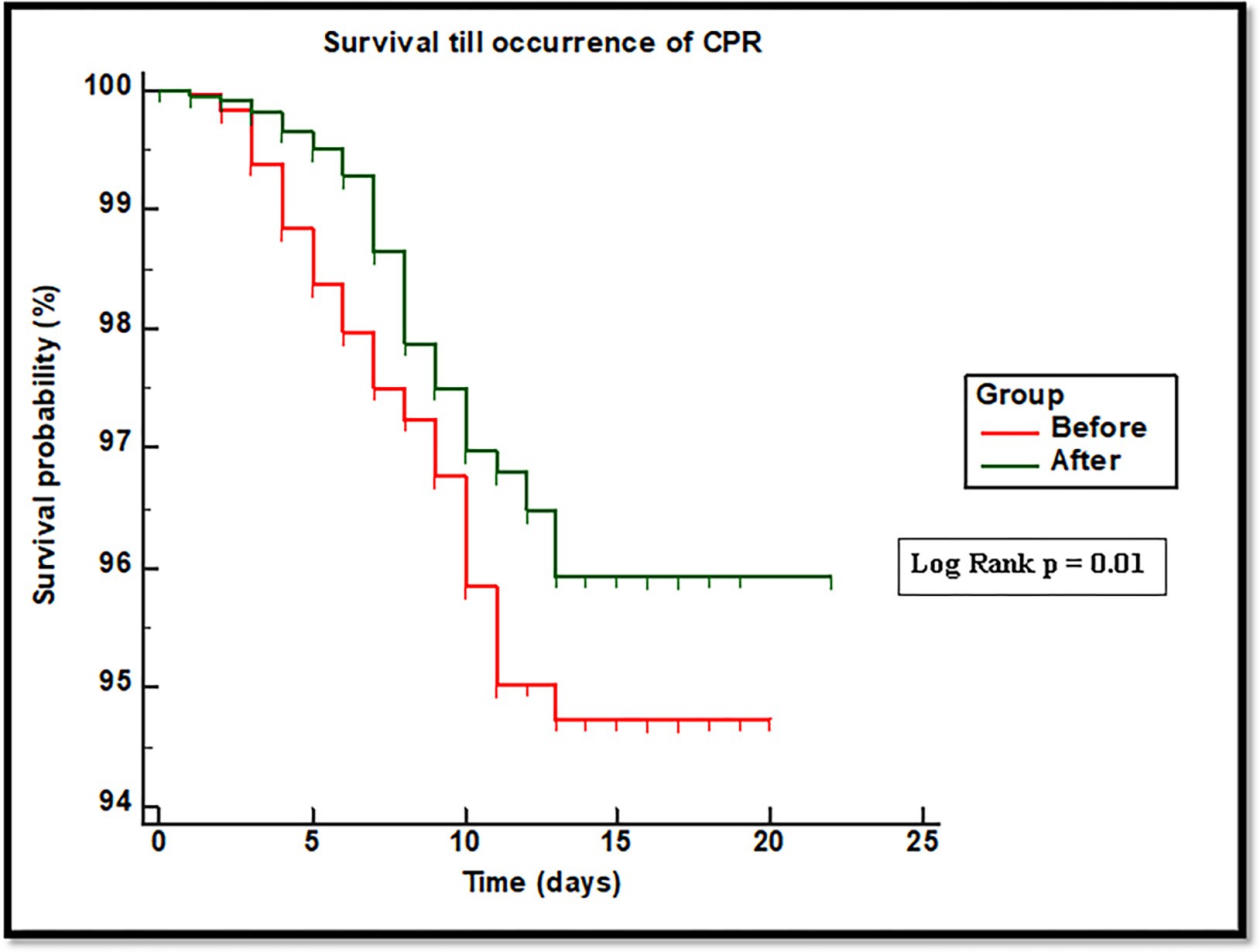
Survival till occurrence of CPR.

The logistic regression model of CPR prediction initially included variables of age, gender, discharge from ICU, MEWS, number of RRT activations, diagnostic category, and the variable of interest (“Before” or “After” group) (S2 Table). The backward deletion method retained three variables in the final model: age (odds ratio (OR) = 1.14 [95% CI: 1.1–1.2]; p < 0.001), number of RRT activations (OR = 0.92 [95% CI: 0.9–0.95]; p < 0.001), and “After” group (OR = 0.67 [95% CI: 0.46–0.99]; p = 0.04). The model was well fitted (Hosmer–Lemeshow test p = 0.12), correctly classified 97.3% of the data, and had an area under the curve of 71% (S3 Fig). A linear relationship between continuous predictors and log CPR was established by Box–Tidwell test p values > 0.05, and there was no collinearity between continuous predictors (correlation coefficient r between age and number of activations = -0.002, p = 0.9). The significantly lower CPR incidence and rate in the “After” group was supported by a significant p value of the log-rank test (p = 0.01) of the depicted Kaplan–Meier curve in the sensitivity analysis (Fig 2).

## Discussion

In this study, we found that applying Masimo Patient SafetyNet^TM^ in medical wards was associated with lower incidence and rate of CPR, higher CPR success rate, shorter LOS, lower hospital mortality, and more RRT activation. The significant predictors of cardiopulmonary arrest and CPR were age (OR = 1.14), number of RRT activations (OR = 0.92), and connection to SafetyNet (OR = 0.67). These results indicate that as age increased by 1 year, the odds of CPR increased by 14%. As the number of RRT activations increased by 1 day, the odds of CPR decreased by 8%, and being in the “After” group decreased the odds of CPR by 33%. These improvements in patient outcomes may be attributed to the timely recognition of worsening vital signs in deteriorating patients.

Since the introduction of RRTs early in the 2000s, several studies have questioned their efficacy, showing no improvement in CPR rates, unplanned ICU admission, average LOS, or hospital mortality, including the MERIT study [23]. This may be explained by the dependence of the RRT in its early models on activation by ward staff, who had to assess the patients and calculate warning scores manually. It follows that if this activation was delayed, the intervention of the RRT would also be delayed and possibly ineffective. What might support this hypothesis is that when the systems evolved to the automated calculation of warning scores and electronic prompts of activation, the results of studies, including this study, almost consistently showed significant improvement, particularly in CPR rates, unplanned ICU admission, LOS, and hospital mortality [13, 16, 17], even when the system still required manual input such as the respiratory rate or level of consciousness. This improvement was also reflected in systematic reviews [1] despite the inevitable differences between studies (in terms of design, population, and RRT structure), thus yielding high heterogeneity percentages, which cast a shadow over those benefits. Similarly, an increased number of activations could also be generated by the severity of the patients, recognized early by the automated system and perhaps not recognized by the healthcare provider, or the opposite, errors in the evaluation of the monitoring system that the healthcare provider could then refute.

One of the most commonly reported outcomes of automated RRT systems is the increase in the number of RRT activations [16, 24], as was the case in our study, since there is no human clinical judgment of the patient’s condition, and the activation is entirely based on numerical values. Undoubtedly, this imposes a burden on team members and increases their workload. However, from another perspective, frequent checks by the team may be advantageous to patients, as their condition gets more frequently re-evaluated, and their management is regularly updated. We demonstrated this benefit in our study as the reduced odds of CPR with increased RRT activation, also shown by others [13, 24], possibly because of the forward progression of management and prevention of deterioration. Notably, the high number of activations and low rates of unplanned admissions indicate a high success rate of RRT management. The finding of increased number of RRT visits may be an indication to reallocate more resources and workforce to RRTs by hospital administrations and policymakers.

ICU admissions were similar in both groups, probably because the need to admit or re-admit patients to the ICU was mandated by the complexity and criticality of their condition, which is beyond the scope of simple interventions offered by the RRT and would have eventually occurred regardless of the team, particularly for medical diagnoses. Many studies that reported a significant reduction in ICU admission and RRT activations included surgical patients [25, 26], who are known to have the most complications in the first three postoperative days [26]. However, studies including both surgical and medical patients have failed to demonstrate such a reduction [27, 28]. A clinical focus review by Michard et al. [29] concluded that continuous surveillance might reduce ICU admissions; however, the review mainly included studies on postoperative patients, leaving them to question which type of patients may benefit from such interventions. Although the “After” period had significantly lower hospital mortality in the medical wards, we cannot claim that this was entirely because of the intervention, as excluded surgical patients were not considered; however, the intervention may have been a contributing factor.

The CPR success rate was notably higher in the “After” group. Although this result cannot be directly attributed to the monitoring system, we speculate that it may have indirectly contributed to it. Having the patient under surveillance by the RRT would probably have decreased the workload of the ward staff; in addition, frequent evaluation and management by the RRT may have caused the precipitating factor of arrest to be easily and quickly reverted. Moreover, the participation of an intensivist and experienced critical care nurses in the CPR could also be a contributing factor. However, this hypothesis remains to be confirmed.

This study demonstrated the benefits of applying the Masimo Patient SafetyNet^TM^ system for automated MEWS calculation and RRT activation. By demonstrating reduced CPR incidence and rate, improved CPR success rate, and reduced hospital LOS and mortality, although coupled with an increased number of RRT activations, we showed the benefits of Tele-RRT in medical patients, who could possibly be even more complex than surgical patients because of comorbidities and advanced age [30]. However, the inclusion of two manual inputs (temperature and LOC) limits the complete automation of the system. Our study enrolled approximately 4500 patients and extended for 16 months. We ranked it among medium-to-large studies in terms of size and duration. However, further studies are needed to evaluate the success of Tele-RRT systems in surgical wards and mixed settings.

Our study has several limitations. The “Before” and “After” designs lack randomization, which subjects the study to possible biases and unmeasured confounders. We included all eligible patients within the study period; therefore, we did not perform any sample size or power calculations. However, our results indicate that the study is adequately powered for the primary outcome of CPR occurrence, as to detect the observed difference between both groups as significant with a 5% type I error rate and 80% power, 2200 patients per group are required; however, for the outcome of CPR rate, our study was underpowered, since the required sample size would be approximately 14000 patients per group. We lacked data on severity scores; however, we included MEWS at the time of RRT activation, which was comparable between the groups. We did not account for total hospital mortality, as was customary in similar studies, since the study did not include hospital wards; however, a reduction in hospital mortality in medical wards was shown. The exclusion of surgical patients is also a limitation as we cannot generalize our results to that group. The study was conducted during the COVID-19 pandemic; however, we did not differentiate between COVID-19 positive and negative patients, which may have yielded different results. We only considered the diagnostic category when other confounding factors, such as comorbidities, could have been imbalanced between the study groups. Furthermore, each group was quite heterogeneous, including different diagnoses with different severities, a situation in which stratification may have been warranted. However, we did not perform stratification because of the small number of events in addition to an under-fitted logistic regression model. This was a single-center study, reflecting the management in only one hospital, which limits the generalizability of its results to the entire region or country. We recommend further studies on surgical or a mix of patients with a design that avoids comparison with historical controls while observing ethical issues. Observational studies that use matching techniques may be an option.

## Conclusion

Automated activation of the RRT by Masimo Patient SafetyNet^TM^ applied to medical ward patients significantly reduced CPR events and rates, reduced hospital length of stay, and increased the number of RRT activations. There was no difference in the ICU admission rates. Further evaluation of the system in surgical wards and mixed settings was conducted.

## Supporting information

S1 Table Modified Early Warning Score. (DOCX)

S2 Table Full logistic regression model. (DOCX)

S1 Fig Medical diagnoses of both groups. (DOCX)

S2 Fig Triggers of RRT activation. (DOCX)

S3 Fig ROC curve of predictive ability of logistic regression model. (DOCX)
