# Correction: Tele-Rapid Response Team (Tele-RRT): The effect of implementing patient safety network system on outcomes of medical patients–A before and after cohort study

**DOI:** 10.1371/journal.pone.0307983

**Published:** 2024-07-24

**Authors:** Ahmed N. Balshi, Mohammed A. Al-Odat, Abdulrahman M. Alharthy, Rayan A. Alshaya, Hanan M. Alenzi, Alhadzia S. Dambung, Huda Mhawish, Saad M. Altamimi, Waleed Th. Aletreby

The timeframe in the “After Period” rectangle in Fig 1 is incorrect. It should have been September 1, 2020 to April 30, 2021. Please see the correct Fig 1 here.

**Fig 1:**
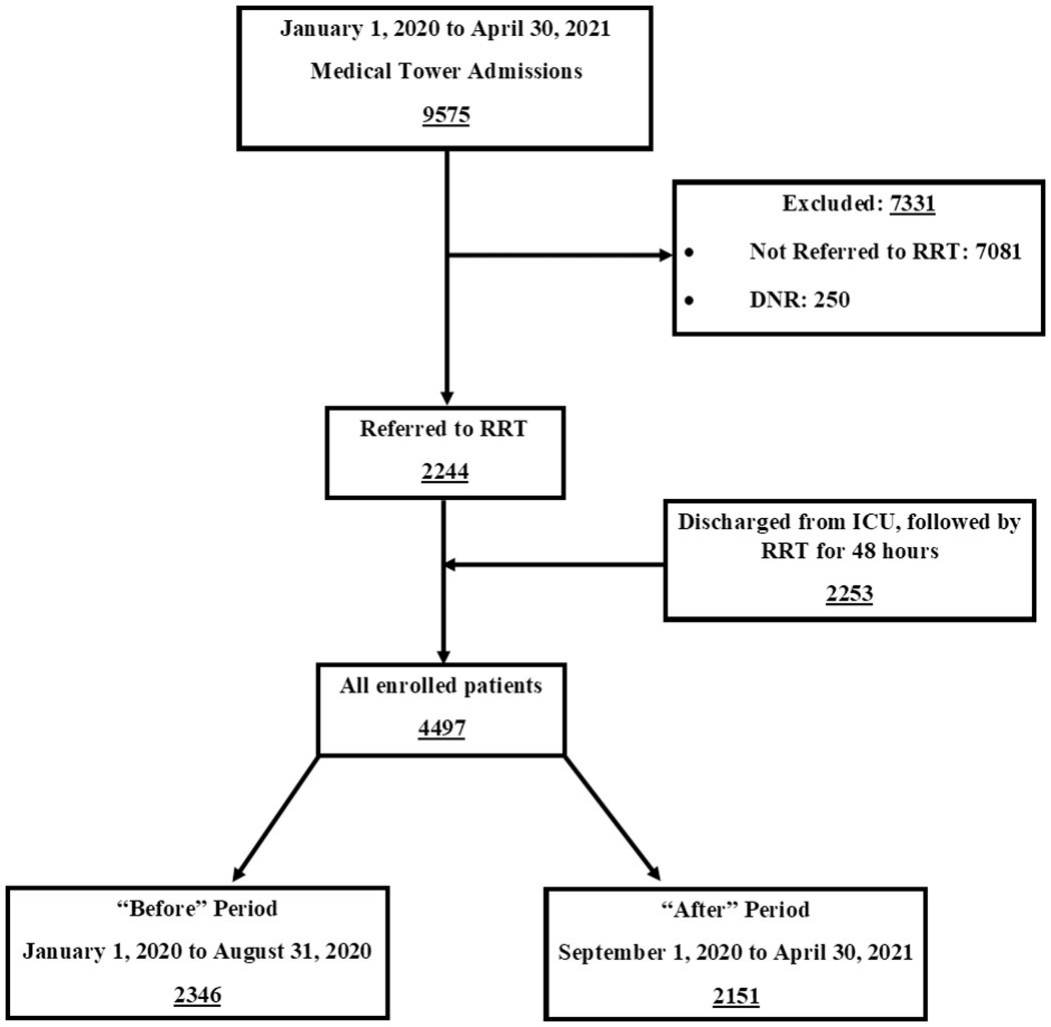
Patients’ enrollment flow diagram.
