# Supplementary material for: Tele-Rapid Response Team (Tele-RRT): The effect of implementing patient safety network system on outcomes of medical patients–A before and after cohort study
Source: PLoS One. 2022 Nov 22;17(11):e0277992. doi: 10.1371/journal.pone.0277992 (PMC9681095; doi:10.1371/journal.pone.0277992)
Supplement: S1 Table — (DOCX) [file pone.0277992.s001.docx]

**S1 Table 1: Modified Early Warning Score:**

| PHYSIOLOGICAL PARAMETERS | 3 | 2 | 1 | 0 | 1 | 2 | 3 |
| --- | --- | --- | --- | --- | --- | --- | --- |
| Temperature |  | **< 35.0** | **35.1-36.0** | **36.1-38.0** | **38.1-39.0** | **> 39.1** |  |
| Systolic Blood Pressure | **< 90** | **91-100** | **101-110** | **111-200** |  | **> 200** | **> 220** |
| Heart Rate | **< 40** |  | **41-50** | **51-100** | **101-110** | **111-130** | **> 131** |
| Respiratory Rate | **< 8** |  | **9 -11** | **12-20** |  |  |  |
| Oxygen Saturation | **< 91** | **92-93** | **94-95** | **> 96** |  |  |  |
| Any Supplemental Oxygen |  | **YES** |  | **NO** |  |  |  |
| Level of Consciousness |  |  |  | **A** | **V** | **P** | **U** |

A = Alert, V = Reacts to voice, P = Reacts to pain, U = Unconscious.
