# Supplementary material for: Tele-Rapid Response Team (Tele-RRT): The effect of implementing patient safety network system on outcomes of medical patients–A before and after cohort study
Source: PLoS One. 2022 Nov 22;17(11):e0277992. doi: 10.1371/journal.pone.0277992 (PMC9681095; doi:10.1371/journal.pone.0277992)
Supplement: S2 Table — (DOCX) [file pone.0277992.s002.docx]

**S1 Table 2: Full Logistic Regression Model:**

| **Variable** | **Odds Ratio** | **95% CI** | **P value** |
| --- | --- | --- | --- |
| **Age (years)** | 1.14 | 1.1 – 1.2 | < 0.001 |
| **Gender**  **(Reference: Male)** | 0.99 | 0.7 – 1.4 | 0.99 |
| **Group**  **(Reference: Before)** | 0.67 | 0.46 – 0.99 | 0.04 |
| **Discharged from ICU** | 0.95 | 0.66 – 1.4 | 0.8 |
| **Number of Activations** | 0.92 | 0.9 – 0.95 | < 0.001 |
| **MEWS** | 0.99 | 0.8 – 1.2 | 0.9 |
| **Diagnostic Category**  **(Reference: Hematology/Oncology)**  **Medical**  **Nephrology**  **Neurology**  **Pulmonology** | 0.92  0.8  0.6  0.96 | 0.4 – 2.4  0.3 – 2.1  0.2 – 1.7  0.3 – 2.8 | 0.9  0.6  0.3  0.9 |
