# Supplementary material for: Tele-Rapid Response Team (Tele-RRT): The effect of implementing patient safety network system on outcomes of medical patients–A before and after cohort study
Source: PLoS One. 2022 Nov 22;17(11):e0277992. doi: 10.1371/journal.pone.0277992 (PMC9681095; doi:10.1371/journal.pone.0277992)
Supplement: S1 Fig — (DOCX) [file pone.0277992.s003.docx]

**S1 Figure 1: Medical diagnoses by groups:**

**
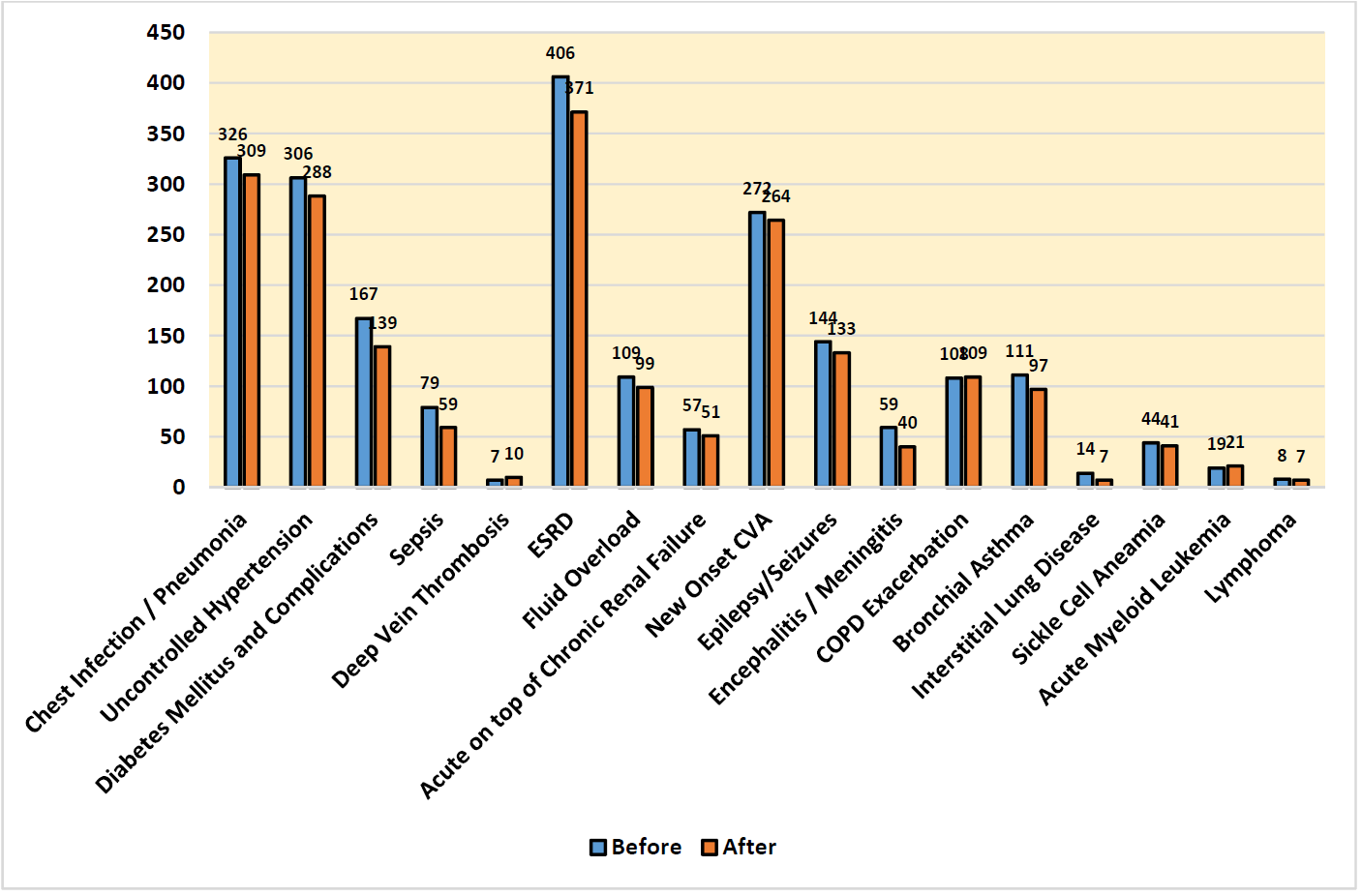
**

ESRD = End stage renal disease, CVA = cerebro-vascular accident, COPD = chronic obstructive pulmonary disease.
