# Supplementary material for: Tele-Rapid Response Team (Tele-RRT): The effect of implementing patient safety network system on outcomes of medical patients–A before and after cohort study
Source: PLoS One. 2022 Nov 22;17(11):e0277992. doi: 10.1371/journal.pone.0277992 (PMC9681095; doi:10.1371/journal.pone.0277992)
Supplement: S2 Fig — (DOCX) [file pone.0277992.s004.docx]

**S1 Figure 2: Triggers of activation of RRT:**

Numbers presented are for triggered activation only (due to MEWS ≥ 5). Visits to patients by RRT may not be triggered by high MEWS (Patients discharged from the ICU in the first 48 hours after discharge).
