# Supplementary material for: Tele-Rapid Response Team (Tele-RRT): The effect of implementing patient safety network system on outcomes of medical patients–A before and after cohort study
Source: PLoS One. 2022 Nov 22;17(11):e0277992. doi: 10.1371/journal.pone.0277992 (PMC9681095; doi:10.1371/journal.pone.0277992)
Supplement: S3 Fig — (DOCX) [file pone.0277992.s005.docx]

**S1 Figure 3: ROC curve of predictive ability of Logistic Regression Model**
